# Supplementary material for: Comparative genomics provides new insights into the diversity, physiology, and sexuality of the only industrially exploited tremellomycete: Phaffia rhodozyma
Source: BMC Genomics. 2016 Nov 9;17:901. doi: 10.1186/s12864-016-3244-7 (PMC5103461; doi:10.1186/s12864-016-3244-7)
Supplement: Additional file 6: — List of orphan genes with links to PFAM (related to Additional file 1: Table S1). (ZIP 1428 kb) [file 12864_2016_3244_MOESM6_ESM.zip › BLAST_HTML_FTR/G02913_P.html]

BLAST Search Results


```
BLASTP 2.2.27+


Reference:
Stephen F. Altschul, Thomas L. Madden, Alejandro A. Schäffer,
Jinghui Zhang, Zheng Zhang, Webb Miller, and David J. Lipman (1997),
"Gapped BLAST and PSI-BLAST: a new generation of protein database
search programs", Nucleic Acids Res. 25:3389-3402.


Reference for
composition-based statistics:
Alejandro A. Schäffer, L. Aravind, Thomas L. Madden, Sergei
Shavirin, John L. Spouge, Yuri I. Wolf, Eugene V. Koonin, and
Stephen F. Altschul (2001), "Improving the accuracy of PSI-BLAST
protein database searches with composition-based statistics and
other refinements", Nucleic Acids Res. 29:2994-3005.


Database: nr
           71,551,133 sequences; 26,053,659,533 total letters


Query= G02913_P

Length=285
                                                                      Score     E
Sequences producing significant alignments:                          (Bits)  Value

emb|CED83470.1|  hypothetical protein [Xanthophyllomyces dendrorh...   581    0.0  
emb|CED82336.1|  hypothetical protein [Xanthophyllomyces dendrorh...  43.5    0.14 
ref|XP_001697173.1|  26S proteasome regulatory subunit [Chlamydom...  40.4    2.4  


 >emb|CED83470.1| hypothetical protein [Xanthophyllomyces dendrorhous]
Length=284

 Score =  581 bits (1497),  Expect = 0.0, Method: Compositional matrix adjust.
 Identities = 284/284 (100%), Positives = 284/284 (100%), Gaps = 0/284 (0%)

Query  1    MGTACWLDGDNPWSSSLDNRGQPCKIYGTVDYIVASVLGGILVVILIALGLGWLIQHSKK  60
            MGTACWLDGDNPWSSSLDNRGQPCKIYGTVDYIVASVLGGILVVILIALGLGWLIQHSKK
Sbjct  1    MGTACWLDGDNPWSSSLDNRGQPCKIYGTVDYIVASVLGGILVVILIALGLGWLIQHSKK  60

Query  61   ASRFRAFWNQSGDEDLEMKTHHHRLINTSEVERHLYDPPMSSAGSVRSGKSKRSSMVPMG  120
            ASRFRAFWNQSGDEDLEMKTHHHRLINTSEVERHLYDPPMSSAGSVRSGKSKRSSMVPMG
Sbjct  61   ASRFRAFWNQSGDEDLEMKTHHHRLINTSEVERHLYDPPMSSAGSVRSGKSKRSSMVPMG  120

Query  121  VSTASSRKSKTGNTGTANARSPLSGWREGDDITSVPNLYSLGGLNTSTSSLSNRSPSPSS  180
            VSTASSRKSKTGNTGTANARSPLSGWREGDDITSVPNLYSLGGLNTSTSSLSNRSPSPSS
Sbjct  121  VSTASSRKSKTGNTGTANARSPLSGWREGDDITSVPNLYSLGGLNTSTSSLSNRSPSPSS  180

Query  181  YPEQVKTLLSPAPSSFLSPPSQYSPYAPSTEYEPSVAAYPIGSPYEQRENQPGSVFRYGM  240
            YPEQVKTLLSPAPSSFLSPPSQYSPYAPSTEYEPSVAAYPIGSPYEQRENQPGSVFRYGM
Sbjct  181  YPEQVKTLLSPAPSSFLSPPSQYSPYAPSTEYEPSVAAYPIGSPYEQRENQPGSVFRYGM  240

Query  241  TEGSNPAVIPGDIGTRPYSPGLYQSQFDDHYRDDRGYTNHQYRR  284
            TEGSNPAVIPGDIGTRPYSPGLYQSQFDDHYRDDRGYTNHQYRR
Sbjct  241  TEGSNPAVIPGDIGTRPYSPGLYQSQFDDHYRDDRGYTNHQYRR  284


>emb|CED82336.1| hypothetical protein [Xanthophyllomyces dendrorhous]
Length=293

 Score = 43.5 bits (101),  Expect = 0.14, Method: Compositional matrix adjust.
 Identities = 24/67 (36%), Positives = 40/67 (60%), Gaps = 5/67 (7%)

Query  53   WLIQHSKKASRFRAFWNQSGDEDL---EMKTHHHRLINTSEVERHLYDPPMSSAGSVRSG  109
            WLI+HS++ +RF+AFWNQS    L   +    +   +N S+V   LYD P +S+ +  +G
Sbjct  16   WLIRHSRQVNRFQAFWNQSASLPLGPADEPGAYEMKVNLSDVS--LYDQPSASSYTSPTG  73

Query  110  KSKRSSM  116
             ++ S +
Sbjct  74   GNRTSRI  80


>ref|XP_001697173.1| 26S proteasome regulatory subunit [Chlamydomonas reinhardtii]
 gb|EDP00428.1| 26S proteasome regulatory subunit [Chlamydomonas reinhardtii]
Length=1037

 Score = 40.4 bits (93),  Expect = 2.4, Method: Composition-based stats.
 Identities = 36/165 (22%), Positives = 68/165 (41%), Gaps = 26/165 (16%)

Query  96   YDPPMSSAGSVRSGKSKRSSMVPMGVSTASSRKSK--------TGNTGTANARSPLSGWR  147
            Y PP+++   V+  K+K  + +    + A+ R+ K        TG  GT+ + +  +G  
Sbjct  829  YPPPLTT--EVKEAKAKVPTAILSTTAKAAERQRKKAAEKEKATGGAGTSTSGAAAAGDA  886

Query  148  ---------EGDDITSVPNLYSLGGLNTSTSSLSNRSPSPSSYPEQVKTLLSPAPSSFLS  198
                     EG D        + G  + + +    + P PSSY  +    ++PA + F++
Sbjct  887  KAADAKPAAEGGDKMETDEAAAGGDKDKAAAGKDKKEPEPSSYTLENPARVTPAQARFVA  946

Query  199  PPSQYSPYAPSTEYEPSVAAYPIGSPYEQRENQPGSVFRYGMTEG  243
             P+       +  ++P  A  P       R+ QPG      +T+G
Sbjct  947  LPA-------TGRWQPVRAGGPATGIMVMRDTQPGEPVELVVTQG  984


Lambda      K        H        a         alpha
   0.314    0.131    0.400    0.792     4.96 

Gapped
Lambda      K        H        a         alpha    sigma
   0.267   0.0410    0.140     1.90     42.6     43.6 

Effective search space used: 2093385537376


  Database: nr
    Posted date:  Sep 23, 2015 12:05 AM
  Number of letters in database: 26,053,659,533
  Number of sequences in database:  71,551,133


Matrix: BLOSUM62
Gap Penalties: Existence: 11, Extension: 1
Neighboring words threshold: 11
Window for multiple hits: 40
```
